# The Effect of Faecal Microbiota Transplantation on Cognitive Function in Cognitively Healthy Adults with Irritable Bowel Syndrome: Protocol for a Randomised, Placebo-Controlled, Double-Blinded Pilot Study

**DOI:** 10.3390/mps8040083

**Published:** 2025-08-01

**Authors:** Sara Alaeddin, Yanna Ko, Genevieve Z. Steiner-Lim, Slade O. Jensen, Tara L. Roberts, Vincent Ho

**Affiliations:** 1 School of Medicine, Western Sydney University, Penrith 2751, Australia; yanna.ko@health.nsw.gov.au (Y.K.); s.jensen@westernsydney.edu.au (S.O.J.); tara.roberts@westernsydney.edu.au (T.L.R.); v.ho@westernsydney.edu.au (V.H.); 2 NICM Health Research Institute and Translational Health Research Institute (THRI), Western Sydney University, Westmead 2145, Australia; g.steiner@westernsydney.edu.au; 3 Department of Psychiatry and Psychotherapy, Jena University Hospital, 07743 Jena, Germany; 4 Ingham Institute of Applied Medical Research, Liverpool 2170, Australia

**Keywords:** faecal microbiota transplantation, gastrointestinal microbiome, cognitive function, irritable bowel syndrome, gut–brain axis

## Abstract

Faecal microbiota transplantation (FMT) is an emerging therapy for gastrointestinal and neurological disorders, acting via the microbiota–gut–brain axis. Altering gut microbial composition may influence cognitive function, but this has not been tested in cognitively healthy adults. This randomised, double-blinded, placebo-controlled pilot trial investigates whether FMT is feasible and improves cognition in adults with irritable bowel syndrome (IBS). Participants receive a single dose of FMT or placebo via rectal retention enema. Cognitive performance is the primary outcome, assessed using the Cambridge Neuropsychological Test Automated Battery (CANTAB). Secondary outcomes include IBS symptom severity and mood. Tertiary outcomes include microbiome composition and plasma biomarkers related to inflammation, short-chain fatty acids, and tryptophan metabolism. Outcomes are assessed at baseline and at one, three, six, and twelve months following treatment. We hypothesise that FMT will lead to greater improvements in cognitive performance than placebo, with benefits extending beyond practice effects, emerging at one month and persisting in the long term. The findings will contribute to evaluating the safety and efficacy of FMT and enhance our understanding of gut–brain interactions.

## 1. Introduction

Faecal microbiota transplantation (FMT) is a gut-microbiome-altering therapy used as a standardised treatment for recurrent *Clostridioides difficile* infections (rCDI) when antibiotics fail. FMT has been demonstrated to be generally safe and well tolerated [1]. Recent research suggests that FMT might be a promising treatment for various conditions beyond rCDI, including inflammatory bowel disease, cirrhosis, and neurological conditions like Parkinson’s disease and dementia [1].

In adults with neurodegenerative diseases, an aberrant microbiome profile has been found, suggesting a link between neurodegeneration and changes in gut microbial composition [2]. FMT may influence the gut microbiome [3], thereby potentially impacting the microbiota–gut–brain axis, a multi-pathway system allowing for bidirectional communication between the brain and the gut and its resident micro-organisms [4]. This interaction may contribute to cognitive enhancement in patients with neurological conditions [5,6,7].

**Figure 1:**
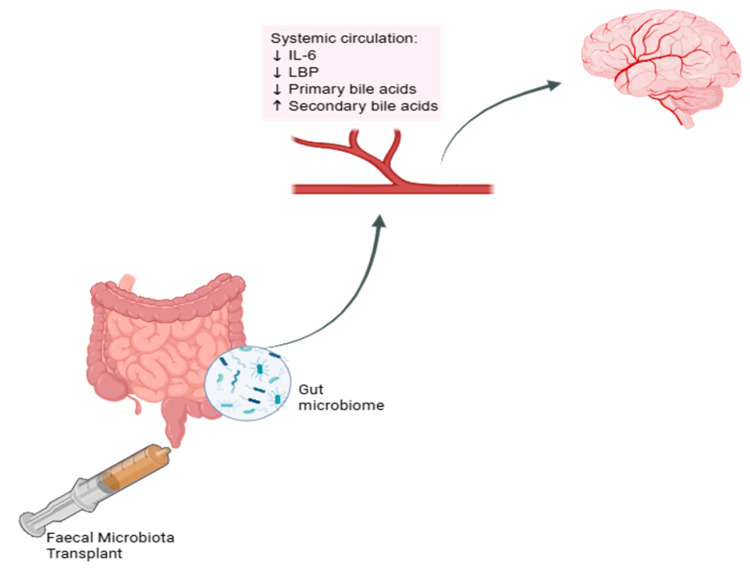
Effect of FMT on systemic circulation markers. Schematic representation of proposed changes in systemic biomarkers in the circulatory system which may influence the brain following faecal microbiota transplantation. IL-6 = interleukin-6, LBP = lipopolysaccharide-binding protein. Created in BioRender [24].

While most clinical studies report changes in the composition of the gut microbiome following FMT [5,7,8], these changes are sometimes accompanied by changes in the blood biomarkers for inflammation and bile acid profile [5,8]. However, the exact mechanisms underlying improvements in cognition remain unclear. FMT studies in humans have noted a reduction in pro-inflammatory cytokine interleukin-6 (IL-6) levels [8,9], a reduction in acute-phase lipopolysaccharide-binding protein [8], and a reduction in primary along with an increase in secondary bile acid levels [9] (see Figure 1).

Several microbial taxa have been linked to cognitive function and gut–brain axis signalling, supporting the biological plausibility of interventions such as FMT. The two key pathways thought to mediate microbiota–brain interactions are short-chain fatty acid (SCFA) production and tryptophan metabolism [4]. Genera such as *Lactobacillus* and *Bifidobacterium* have been associated with better cognitive performance [10,11] and are the key producers of short-chain fatty acids (SCFAs) [12]. Altered SCFA levels have been linked to various neurological disorders [13]. In individuals with IBS, changes in SCFA concentrations following FMT have been shown to persist for over one year [14].

Furthermore, *Lactobacillus reuteri* has been shown to influence the kynurenine pathway [15], a tryptophan-derived metabolic route implicated in cognitive outcomes [16]. More broadly, the gut microbiome has also been shown to modulate the metabolism of tryptophan to kynurenine [17], potentially through modulation of the inflammatory mediators [18] that regulate kynurenine metabolism [19]. An increased kynurenine-to-tryptophan ratio has been associated with cognitive impairment [16], and probiotics have been found to modulate this ratio [20]. Together, these microbial pathways, SCFA production and tryptophan metabolism, represent compelling targets for monitoring in this study, given their potential links to cognitive function and the gut-rain axis.

Preclinical studies further support the notion that FMT modifies bowel microbial composition and inflammation [21]. Notably, transferring FMT from old donor mice into young mice leads to an increase in pro-inflammatory cytokines in the latter, while transferring FMT from young donor mice into old recipient mice decreases circulating pro-inflammatory cytokines [22]. Additionally, in a transgenic Alzheimer’s disease (AD) mouse model (5xFAD), FMT could rescue object recognition performance assessed via the novel object recognition test and could improve spatial memory in a forced alternation Y-maze task. Furthermore, a reduction was observed in cortical amyloid beta after FMT compared to untreated controls [23].

Studies in cognitively healthy individuals have explored the effects of pre- and probiotics on cognitive function and found improvements in memory and processing speed [25]. However, some studies also report declines in cognitive function [26], which implies that microbial changes are not always beneficial in cognitively healthy adults. Beyond bacteria, the gut virome, predominantly consisting of bacteriophages, appears to influence executive function and memory [27]. Furthermore, the composition of the gut mycobiome, the small fungal component of the gut microbiome, has been found to differ in people with mild cognitive impairment compared to healthy human controls [28]. Unlike other microbiome alteration techniques, such as pre- and probiotics, FMT not only targets the bacterial composition but also the gut virome and mycobiome [29], offering a more comprehensive intervention.

To the best of our knowledge, no studies have examined the cognitive effects or safety of FMT in cognitively healthy adults. Given that FMT has been shown to alter the gut bacteriome, virome, and mycobiome [29], we hypothesise that FMT has the potential to modulate cognitive function compared to placebo. Probiotic studies have shown an effect on cognition as early as four weeks following intervention [30]. Therefore, we chose our first two follow-up visits to be at one month and three months following intervention. To investigate the potential long-term effects on the microbiome, blood markers, and cognitive function, we included follow-up visits at six months and twelve months.

Our study looks at a subsample of participants who receive a single 60 g dose of FMT or placebo via rectal retention enema as part of a broader investigation into the efficacy of FMT in irritable bowel syndrome (IBS), a prevalent gut–brain axis disorder [31]. The first-line treatment of care for IBS is dietary change, especially the low fermentable, oligosaccharide-, disaccharide-, monosaccharide-, and polyol (FODMAP) diet [32], in addition to over-the-counter medication, such as laxatives and loperamide [33]. FODMAPs are short-chain carbohydrates, which can exacerbate IBS symptoms, and restricting their intake has been found to improve symptoms [34]. While IBS symptoms include abdominal pain accompanied by changes in stool frequency or form [31], individuals with IBS have been found to perform similarly to healthy controls on neuropsychological screenings of cognitive function [35], making this a suitable cohort for investigating subtle cognitive modulation.

Only participants who do not significantly improve following the low FODMAP diet will be included. The selected FMT dose has shown efficacy in IBS [36], and rectal delivery was chosen for its superior effectiveness in rCDI [37] and ulcerative colitis [38], with enemas being less risky than colonoscopy [39]. Continued use of standard IBS medications during the trial is permitted, justifying the use of a placebo control.

This article was previously presented as a meeting abstract at the Australasian Neuroscience Society 2024 Annual Scientific Meeting on the 4th of December 2024.

## 2. Experimental Design

Study visits will take place at Macarthur Clinical School in Campbelltown and the NICM Health Research Institute in Westmead. The intervention will be administered at Campbelltown Private Hospital. All sites are affiliated with Western Sydney University. This study is a sub-study of a larger clinical trial registered with the Australian New Zealand Clinical Trial Registry (ACTRN12623000353695) in accordance with the World Health Organization Trial Registration Data Set (see Supplementary File S1). While the main trial focuses on IBS symptom severity, the present sub-study investigates cognitive outcomes as the primary endpoint.

Participants will be eligible for inclusion if they are aged 18 years or older and meet the ROME IV criteria for IBS, defined as recurrent abdominal pain occurring at least once per week over the past three months, accompanied by at least two of the following: pain related to defaecation, changes in stool frequency, or changes in stool form. Normal cognitive function is defined as a score of ≥26 in the Montreal Cognitive Assessment (MoCA). Only individuals who fail to respond to a dietician-guided low FODMAP diet—defined as a reduction of less than 50 points on the IBS Symptom Severity Score (IBS-SSS) after 21 days—will be included.

The exclusion criteria include gastrointestinal or neurological comorbidities, MoCA < 26, significant symptom reduction after the low FODMAP diet (≥50 points), pregnancy or lactation, a history of abdominal surgery (except appendectomy, cholecystectomy, caesarean section, or hysterectomy), severe psychiatric illness, active gastroenteritis, recent use of antibiotics or probiotics (within eight weeks), use of IBS-specific medications within the past three months (except polyethylene glycol or loperamide), and substance misuse.

As part of the sub-study, we aim to recruit 20 participants, with 10 allocated to the FMT group and 10 to the placebo group. The first 20 participants enrolled in the overarching trial will be recruited into this sub-study and will complete additional cognitive tests and questionnaires at all study visits. Recruitment will take place through the gastroenterology outpatient clinic at Campbelltown Hospital, a Meta-advertisement campaign run by Western Sydney University, and a university-maintained database of participants from a previous IBS clinical trial.

To confirm eligibility, potential participants will be screened for cognitive health using the MoCA and then referred to a study dietitian for a one-on-one, one-hour education session on the low FODMAP diet. Participants will then follow the diet for 21 days under dietitian supervision. Adherence is monitored through daily food diaries, submitted weekly and reviewed via phone consultation. Participants are encouraged to contact the study team with any diet-related questions. Adherence is calculated as the percentage of meals compliant with the low FODMAP guidelines, based on food diaries, and it is considered successful if more than 80% of meals meet the criteria. The IBS-SSS will be completed before starting the diet and on the final day of the diet. If participants achieve less than a 50-point reduction on the IBS-SSS and a MoCA score of 26 or more, they are deemed eligible for participation in this trial.

At the baseline visit, eligible participants will provide informed consent and complete the Cambridge Neuropsychological Test Automated Battery (CANTAB), the Depression Anxiety Stress Scale (DASS-42), IBS-SSS, and the IBS Quality of Life questionnaire (IBS-QoL).

Following baseline assessment, participants will be randomised to receive a single rectal retention enema containing either FMT or placebo using the National Cancer Institute Clinical Trial Randomization Tool, applying a maximum tolerated imbalance of two. The enema will consist of a 60 mL dose and will be administered by a gastroenterologist with the participant lying in the left lateral position, followed by a 20 min monitoring period. FMT and placebo materials will be prepared in advance in accordance with the Australian Therapeutic Goods Administration (TGA) (Therapeutic Goods Order 105/107) guidelines, and syringes will be labelled by a researcher not involved in data collection or intervention delivery to ensure blinding of participants, clinicians, and research staff.

Stool donors will be screened in accordance with TGA guidelines (Therapeutic Goods Order 105) for human intestinal microbiota transplants. Eligible donors are healthy adults with no history of chronic gastrointestinal disorders, infectious diseases, recent antibiotic use, or high-risk behaviours. Screening includes a detailed social and medical history, physical examination, and laboratory testing of both blood and stool samples. Blood tests include screening for HIV-1 and HIV-2, hepatitis A, B, and C, syphilis, HTLV-I/II, and other transfusion-transmissible infections. Stool samples are tested for common enteric pathogens, including *Clostridioides difficile*, *Salmonella*, *Shigella*, *Campylobacter*, *Giardia*, *Cryptosporidium*, and norovirus, as well as for antimicrobial-resistant organisms, such as extended-spectrum beta-lactamase-producing bacteria. Donors are also screened for COVID-19. All screening procedures are repeated every 90 days during the donation period.

**Figure 2:**
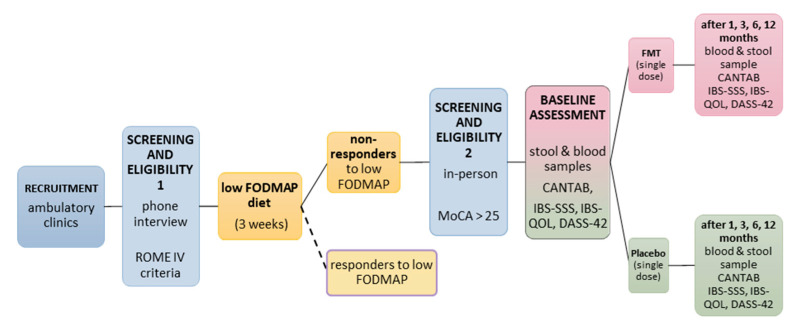
Participant timeline. Screening procedure, diet phase, and cognitive and biomarker assessment at baseline and follow-ups in FMT and placebo groups. Participants are randomised to FMT or placebo, and all involved in data collection and treatment administration are blinded to the group allocation. Blue = recruitment/screening for eligibility; yellow = diet phase; pink = FMT group; green = placebo group.

Participants will return for follow-up assessments at one, three, six, and twelve months following treatment (see Figure 2). At each time point, they will complete all questionnaires and cognitive tests and provide stool and blood samples.

This pilot trial will assess the feasibility of the protocol and provide preliminary estimates of the effect size. Cognitive performance, inflammatory markers, and microbial metabolites will be analysed using mixed-design ANOVA, with group (FMT vs. placebo) as the between-subjects factor and time (baseline, one, three, six, and twelve months) as the within-subjects factor. Planned contrasts will examine within-group changes over time. Microbiome composition will be compared using PERMANOVA with Bray–Curtis dissimilarity, and missing data will be addressed using multiple imputation.

Participants are advised to contact the trial coordinator if they experience any side effects. Although FMT is generally safe and well tolerated, minor adverse events, such as constipation, diarrhoea, or low-grade fever, may occur, typically resolving within 48 h. These will be reviewed by a gastroenterologist and documented in a password-protected file. A serious adverse event is defined according to Australian National Health and Medical Research Council guidelines as any event resulting in death, being life-threatening, requiring or prolonging hospitalisation, causing significant disability, or leading to a congenital anomaly. Serious adverse events will be monitored until resolution.

Due to the low-risk nature of this pilot study and the small sample size, a Data Safety Monitoring Board is not required. Participants in the placebo group who complete all follow-up visits will be offered FMT at the end of the trial, provided blinding is maintained until data collection is complete.

As a pilot study, this trial is not powered to detect small or moderate effects. The main aim is to assess the feasibility, test procedures, and to explore the potential signals of cognitive benefit to inform the design and sample size of a future large-scale trial. While all participants are carefully screened, individual variability in microbiota composition and baseline cognitive performance may affect outcomes. Additionally, the use of a single FMT dose and lack of dietary control following intervention may limit the generalisability of the results.

### 2.1. Outcome Measures

This protocol includes outcome measures across three domains. These outcomes will be analysed to assess feasibility, identify sensitive indicators of cognitive change, and guide the design of future trials.

- Primary outcome: Cognitive performance, assessed using the Cambridge Neuropsychological Test Automated Battery (CANTAB), which includes subtests for memory, attention, processing speed, and executive function.
- Secondary outcomes: Mood, measured using the Depression Anxiety Stress Scale (DASS-42), and IBS symptom severity, measured using the IBS Symptom Severity Score (IBS-SSS). The IBS Quality of Life (IBS-QoL) questionnaire is administered but will not be analysed in this sub-study.
- Tertiary outcomes: Stool-based gut microbiome composition and function (via shotgun metagenomic sequencing and SCFA quantification) and plasma-based biomarkers, including pro-inflammatory cytokines, short-chain fatty acids, and tryptophan metabolites.

### 2.2. Materials

1. ColOff stool collection device (Zymo Research, Irvine, CA, USA, R1101-2-5).
2. DNA/RNA Shield Fecal Collection Tube (Zymo Research, Irvine, CA, USA, R1101).
3. Glycerol, for molecular biology, ≥99.0% (Sigma Aldrich, Burlington, MA, USA, G5516).
4. Phosphate-buffered saline, pH 7.4, sterile-filtered, suitable for cell culture (Sigma Aldrich, Burlington, MA, USA, P4474).
5. Transfer pipette (Sigma Aldrich, Burlington, MA, USA, HS206371C).
6. 60 mL syringes without needle (Terumo, Tokyo, Japan, SS60L).

### 2.3. Equipment

- iPad (7th generation, Apple Inc., Cupertino, CA, USA);
−80 °C freezer (ThermoFisher Scientific, Waltham, MA, USA, TSX60086FA);
- LC-MS system (SCIEX QTRAP 7500);
- Proteomics for cytokines (Olink Target 48 cytokines).

## 3. Procedure

### 3.1. Intervention Preparation

1. Screen potential stool donors according to TGA Therapeutic Goods Order 105 for eligibility.
2. Collect stool in a sterile bucket within 6 h after defaecation.
3. Weigh stool.
4. Mix stool with phosphate-buffered saline and ten per cent pharmaceutical-grade glycerol to reach a ten percent concentration of stool.
5. Draw the resulting faecal suspension into 60 mL syringes labelled according to TGA Therapeutic Goods Order 107.
6. Produce placebo by mixing glycerol and saline solution (ratio 5:1), 5 g fibre, and 2 mL food colouring per litre.
7. Draw the resulting suspension into 60 mL syringes.
8. Cover all syringes in aluminium foil to ensure blinding.
9. Store at −80 °C.

### 3.2. Pre-Treatment Phase (~3 Weeks)

1. Recruit participants, provide study information, and obtain written informed consent (for the consent form, see Supplementary Materials).
2. Conduct MoCA and confirm a score of ≥26.
3. Refer potential participants to study dietician for introduction to low FODMAP diet.
4. Study dietician supervises the diet for three weeks. Adherence is monitored through food diaries and weekly phone calls.
5. Participants complete the IBS-SSS during their first appointment with the dietician and again after 21 days on the diet.
6. Score IBS-SSS.

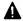
 **CRITICAL STEP** Only invite participants with a <50-point IBS-SSS reduction and a MocA score of >25 to the baseline visit.

### 3.3. Baseline Assessment (~2 h)

1. Administer CANTAB, DASS-42, and IBS-SSS.
2. Draw blood (2 × 9 mL EDTA tubes).
3. Provide participant with a stool sampling kit (see Supplementary File S2).
4. Centrifuge whole blood samples at 4 °C for 10 min to obtain plasma. Aliquot plasma into 1.5 mL Eppendorf tubes and store at −80 °C.

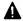
 **CRITICAL STEP** Process whole blood samples within 60 min of collection.

### 3.4. Intervention Visit (~1 h)

1. Thaw FMT or placebo syringes in an ice bath for two hours.
2. Collect stool sample from participant and store at −80 °C.
3. Provide participant with a fresh stool sampling kit for use before the follow-up visit.
4. Administer 60 mL of the assigned treatment via rectal retention enema while the participant lies in a left lateral position.

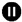
 **PAUSE STEP** Ask participants to remain in the lateral position for 20 min under medical supervision to ensure adherence.

### 3.5. Follow-Up Visits (1, 3, 6, 12 Months After Intervention)

Repeat questionnaires and cognitive tests (CANTAB, DASS-42, IBS-SSS).

Collect stool samples (self-collected at home within 24 h prior to visit) and draw blood samples on-site (2 * 9 mL EDTA tubes).

Centrifuge blood at 4 °C for 10 min to obtain plasma. Aliquot into 1.5 mL Eppendorf tubes and store at –80 °C.

Store all stool and plasma samples at –80 °C until analysis. Plasma will be analysed for pro-inflammatory cytokines via ELISA and for tryptophan metabolites and short-chain fatty acids via liquid chromatography–mass spectrometry (LC-MS).

## 4. Expected Results

We expect cognitive performance in the placebo group to remain stable or show minor improvements over time due to practice effects. In contrast, participants receiving FMT may exhibit changes in cognitive function, including improvement or variability, reflecting the exploratory nature of this intervention in a cognitively healthy population. The expected cognitive changes may include faster reaction times, improved memory accuracy, or enhanced executive functioning.

Biological markers are anticipated to remain unchanged in the placebo group but may shift in the FMT group, consistent with microbiome modulation. These may include increased gut microbial diversity, reduced circulating pro-inflammatory cytokines, such as IL-6 and TNF-α, and shifts in short-chain fatty acid and tryptophan metabolite concentrations.

If the protocol is unsuccessful, the cognitive scores are expected to remain unchanged or decline slightly, and biological markers would show no consistent or directional changes over time. A full description of the outcomes assessed (including cognitive, psychological, microbial, and biochemical variables) is provided in Section 2 (Experimental Design).

## Data Availability

De-identified data supporting the findings of this study will be made available upon reasonable request from the corresponding author. Microbiome data will be deposited in a public repository following study completion. Access to participant-level data is restricted due to ethical and privacy considerations.

## Supplementary Materials

The following supporting information can be downloaded at: https://www.mdpi.com/article/10.3390/mps8040083/s1. Supplementary File S1: Consent form; Supplementary File S2: Stool sample collection instructions; Supplementary File S3: World Health Organization Trial Registration Data Set.

## Abbreviations

The following abbreviations are used in this manuscript:

ACTRN	Australian New Zealand Clinical Trials Registry Number
AD	Alzheimer’s Disease
CANTAB	Cambridge Neuropsychological Test Automated Battery
DASS-42	Depression Anxiety Stress Scale (42-item version)
ELISA	Enzyme-Linked Immunosorbent Assay
FMT	Faecal Microbiota Transplantation
IBS	Irritable Bowel Syndrome
IBS-QoL	IBS Quality of Life Instrument
IBS-SSS	IBS Symptom Severity Score
IL-6	Interleukin-6
LBP	Lipopolysaccharide-Binding Protein
LC-MS	Liquid Chromatography–Mass Spectrometry
MoCA	Montreal Cognitive Assessment
OTS	One-Touch Stockings of Cambridge
PAL	Paired Associates Learning
PRM	Pattern Recognition Memory
rCDI	Recurrent Clostridioides difficile Infection
RTI	Reaction Time
SCFA	Short-Chain Fatty Acid
SPIRIT	Standard Protocol Items: Recommendations for Interventional Trials
SWM	Spatial Working Memory
TGA	Therapeutic Goods Administration
TGO	Therapeutic Goods Order
VRM	Verbal Recognition Memory
