# Supplementary material for: The Effect of Faecal Microbiota Transplantation on Cognitive Function in Cognitively Healthy Adults with Irritable Bowel Syndrome: Protocol for a Randomised, Placebo-Controlled, Double-Blinded Pilot Study
Source: Methods Protoc. 2025 Aug 1;8(4):83. doi: 10.3390/mps8040083 (PMC12388800; doi:10.3390/mps8040083)
Supplement: Supplementary file 1 [file mps-08-00083-s001.zip › mps-3652594-supplementary.pdf]

Supplementary File S1

Consent Form

Project Title: Faecal microbiota transplantation versus low FODMAP diet in the treatment of irritable bowel syndrome. This study has been approved by the Human Research Ethics Committee at Western Sydney University. The ethics reference number is H14709.

I hereby consent to participate in the above-named research project and understand that I can be randomised into either the faecal microbiota transplantation group or the placebo group.

I acknowledge that

- I have read the participant information sheet (or, where appropriate, have had it read to me) and have been given the opportunity to discuss the information and my involvement in the project with the researcher/s.
- The procedures required for the project and the time involved have been explained to me, and any questions I have about the project have been answered to my satisfaction.

I consent to

☒ *Being randomised to either receiving faecal microbiota transplantation or a placebo treatment;*
☒ *Completing a baseline health questionnaire and cognitive assessment;*
☒ *Having my medical history taken or obtained from my existing medical records;*
☒ *Providing stool samples for microbiome analysis;*
☒ *Providing blood samples for analysis;*
☒ *Completing health questionnaires and cognitive screenings at each follow-up visit;*
☒ *Providing stool samples for additional analysis, including use in zebrafish research to investigate biological mechanisms.*

Data publication, re-use, and storage

This project seeks consent for the data provided to be used in any other projects in the future.
To make re-use of the data possible, they will be stored under Western Sydney University's Open Access Policy.

I understand that in relation to the publication of the data

☐ **My involvement is confidential, and the information gained during the study may be published, but no information about me will be used in any way that reveals my identity.**
☐ **The researchers intend to make the non-identified data from this project available for other research projects.**
☐ **I can withdraw from the study at any time without affecting my relationship with the researcher/s and any organisations involved, now or in the future.**

Signed:
Name:
Date:

Post-trial access to the trial interventions

If you would like to be notified about newly available access to the study's interventions, please provide an email address or phone number that you would like to be contacted through for this information.

What if I have a complaint?

If you have any complaints or reservations about the ethical conduct of this research, you may contact the Ethics Committee through Research Engagement, Development, and Innovation (REDI) on Tel. +61-(0)2-4736-0229 or email humanethics@westernsydney.edu.au.
Any issues you raise will be treated in confidence and investigated fully, and you will be informed of the outcome.

Supplementary File S2

Stool sample collection instructions

Stool Collection Equipment

1 Pair of latex gloves
1 ColOff specimen collector
1 Transfer pipette
1 DNA/RNA Shield™ Fecal Collection Tube (brown tube top)
1 Biohazard zip lock bag
1 Stool collection instruction
1 Waste bag

Stool Collection—Instruction

Stool deposit

1. Empty the bladder if required. The stool sample should not be contaminated with urine.
2. Remove the ColOff® from its packaging and unfold it.
3. Place the ColOff® sleeve around your toilet seat like a sleeve, with the hole facing upwards towards the ceiling. Gently push down the bottom of the sleeve.
4. Deposit your bowel movement into the ColOff® sleeve.
5. Any toilet paper used should not be deposited inside the ColOff® sleeve. Dispose of used toilet paper into the waste bag provided.

Stool collection

6. Put gloves on.
7. Unscrew the cap from the brown top tube with buffer. Ensure the tube is held upright or placed on a stable surface to avoid spilling the tube contents.
8. *For solid stool:* Using the attached spoon (attached to the tube cap), collect a full spoonful of stool and replace the lid and spoon into the collection tube.
9. *For liquid stool:* Use the transfer pipette provided to collect approximately 1 millilitre of liquid stool (1 full pipette) and transfer it to the collection tube.
10. Securely tighten the lid and *shake vigorously* for 1 min.
11. Place the tube into the biohazard zip lock bag.
12. Dispose of the remaining stool in the toilet.
13. The ColOff specimen collector is not flushable. Please dispose of the collector and gloves in the waste bag provided and throw it out in the bin.
14. Wash hands.
15. Place the red biohazard zip lock bag containing the collection tube with your stool sample into the clear zip lock bag and return it to Dr Yanna Ko or Sara Alaeddin within 24 h.

Supplementary File S3

World Health Organization Trial Registration Data Set

**Item**	**Description**
1. Primary registry and trial-identifying number	Australian New Zealand Clinical Trial Registry: ACTRN12623000353695
2. Date of registration in primary registry	05/04/2024
3. Secondary identifying numbers	Ethics Approval H14709
4. Sources of monetary or material support	Western Sydney University
5. Primary sponsor	Western Sydney University
6. Secondary sponsor(s)	N/A
7. Contact for public queries	Sara Alaeddin<br>Email: s.alaeddin@westernsydney.edu.au
8. Contact for scientific queries	Principal Investigator:<br>A/Prof Vincent Ho<br>Email: v.ho@westernsydney.edu.au
9. Public title	Faecal microbiota transplantation for the treatment of irritable bowel syndrome (FMT study)
10. Scientific title	Faecal microbiota transplantation versus low FODMAP diet in the treatment of irritable bowel syndrome
11. Countries of recruitment	Australia
12. Health condition(s) or problem(s) studied	Irritable bowel syndrome
13. Intervention(s)	Single dose (60 mL) of faecal microbiota transplant or placebo delivered via rectal retention enema
14. Key inclusion and exclusion criteria	Inclusion criteria:<br>✓ ≥18 years old;<br>✓ Meets the ROME IV criteria for IBS;<br>✓ No history of concurrent gastrointestinal or neurological diseases;<br>✓ Normal cognitive function (score of >25 on the MoCA);<br>✓ IBS symptom severity not responding to the low fermentable, oligosaccharide-, disaccharide-, monosaccharide-, and polyol (FODMAP) diet.
15. Study type	Method of allocation: Randomised, double-blinded, placebo-controlled<br>Assignment: Parallel purpose: Feasibility, cognitive safety of treatment<br>Phase: Pilot allocation and randomisation: Maximally tolerated imbalance procedure (Tool: NIH Clinical Trial Randomization Tool; https://ctrandomization.cancer.gov, accessed on 03/04/2023)<br>Maximally tolerated imbalance value: 2
16. Date of first enrolment	5 April 2023
17. Target sample size	20
18. Recruitment status	Complete
19. Primary outcome(s)	Cognitive performance measured via CANTAB Reaction Time (processing speed): Latency<br>Verbal recognition memory (verbal memory): Free recall (number of words), immediate and delayed recognition (number of correct and incorrect responses)<br>Pattern recognition memory (visual memory: number and percentage of correct trials, latency)<br>Paired Associates Learning (working memory): Error rate, number of trials required, memory scores, stages completed

20. Key secondary outcome(s)	One-Touch Stockings of Cambridge (executive function): Number of problems solved on first choice, mean choices to correct, mean latency to first choice, mean latency to correct<br>Spatial working memory (working memory): Error rate<br><u>Time point of interest:</u><br>Baseline and all follow-up time points<br><u>Plasma samples</u><br>Olink Target 48 Cytokine<br>Concentrations of<br>• Interleukin-18<br>• Hepatocyte growth factor<br>• C-C motif chemokine 19<br>• C-C motif chemokine 2<br>• Macrophage metalloelastase<br>• Lymphotoxin-alpha<br>• FMS-related tyrosine kinase 3 ligand<br>• Tumour necrosis factor<br>• Interleukin-17A<br>• Interleukin-2<br>• Interleukin-17F<br>• Granulocyte colony-stimulating factor<br>• Interleukin-1 beta<br>• Oxidised low-density lipoprotein receptor 1<br>• Tumour necrosis factor ligand superfamily member 12<br>• C-X-C motif chemokine 10<br>• Vascular endothelial growth factor A<br>• Interleukin-33<br>• Thymic stromal lymphopoietin<br>• Interferon gamma<br>• C-C motif chemokine 4<br>• Pro-transforming growth factor alpha<br>• Interleukin-13<br>• Interleukin-8<br>• C-C motif chemokine 8<br>• Interleukin-6<br>• C-C motif chemokine 13<br>• Granulocyte-macrophage colony-stimulating factor<br>• C-C motif chemokine 7<br>• Interleukin-4<br>• Tumour necrosis factor ligand superfamily member 10<br>• Oncostatin-M<br>• Interstitial collagenase<br>• Pro-epidermal growth factor<br>• Interleukin-7<br>• Interleukin-15<br>• Macrophage colony-stimulating factor 1<br>• C-X-C motif chemokine 9<br>• C-X-C motif chemokine 11<br>• Interleukin-17C<br>• Stromal cell-derived factor 1<br>• Eotaxin<br>• Interleukin-10

- C-C motif chemokine 3
- Interleukin-27

Liquid chromatography–mass spectrometry:
Short-chain fatty acid concentrations in plasma

- Acetic acid
- Butyric acid
- Isovaleric acid
- Propionic acid
- Valeric acid

Tryptophan metabolite concentrations of

- Tryptophan
- Kynurenine

<u>Stool samples</u>
Shotgun metagenomic sequencing:

- • Alpha diversity
- • Beta diversity
- • Metagenomic function profiling

<u>Time points of interest:</u>
Baseline and all follow-up visits
